# Impact of Hospital Characteristics and Governance Structure on the Adoption of Tracking Technologies for Clinical and Supply Chain Use: Longitudinal Study of US Hospitals

**DOI:** 10.2196/33742

**Published:** 2022-05-26

**Authors:** Xiao Zhu, Youyou Tao, Ruilin Zhu, Dezhi Wu, Wai-kit Ming

**Affiliations:** 1 Department of Clinical Pharmacy and Pharmacy Administration School of Pharmacy Fudan University Shanghai China; 2 Department of Information Systems and Business Analytics College of Business Administration Loyola Marymount University Los Angeles, CA United States; 3 Management Science Department Lancaster University Management School Lancaster University Lancaster United Kingdom; 4 Department of Integrated Information Technology College of Engineering and Computing University of South Carolina Columbia, SC United States; 5 Department of Infectious Diseases and Public Health Jockey Club College of Veterinary Medicine and Life Sciences City University of Hong Kong Hong Kong Hong Kong

**Keywords:** radio frequency identification, bar coding, tracking technology adoption, smart hospital, hospital affiliation, governance structure, location, clinical use, supply chain use

## Abstract

**Background:**

Despite the increasing adoption rate of tracking technologies in hospitals in the United States, few empirical studies have examined the factors involved in such adoption within different use contexts (eg, clinical and supply chain use contexts). To date, no study has systematically examined how governance structures impact technology adoption in different use contexts in hospitals. Given that the hospital governance structure fundamentally governs health care workflows and operations, understanding its critical role provides a solid foundation from which to explore factors involved in the adoption of tracking technologies in hospitals.

**Objective:**

This study aims to compare critical factors associated with the adoption of tracking technologies for clinical and supply chain uses and examine how governance structure types affect the adoption of tracking technologies in hospitals.

**Methods:**

This study was conducted based on a comprehensive and longitudinal national census data set comprising 3623 unique hospitals across 50 states in the United States from 2012 to 2015. Using mixed effects population logistic regression models to account for the effects within and between hospitals, we captured and examined the effects of hospital characteristics, locations, and governance structure on adjustments to the innate development of tracking technology over time.

**Results:**

From 2012 to 2015, we discovered that the proportion of hospitals in which tracking technologies were fully implemented for clinical use increased from 36.34% (782/2152) to 54.63% (1316/2409), and that for supply chain use increased from 28.58% (615/2152) to 41.3% (995/2409). We also discovered that adoption factors impact the clinical and supply chain use contexts differently. In the clinical use context, compared with hospitals located in urban areas, hospitals in rural areas (odds ratio [OR] 0.68, 95% CI 0.56-0.80) are less likely to fully adopt tracking technologies. In the context of supply chain use, the type of governance structure influences tracking technology adoption. Compared with hospitals not affiliated with a health system, implementation rates increased as hospitals affiliated with a more centralized health system—1.9-fold increase (OR 1.87, 95% CI 1.60-2.13) for decentralized or independent hospitals, 2.4-fold increase (OR 2.40, 95% CI 2.07-2.80) for moderately centralized health systems, and 3.1-fold increase for centralized health systems (OR 3.07, 95% CI 2.67-3.53).

**Conclusions:**

As the first of such type of studies, we provided a longitudinal overview of how hospital characteristics and governance structure jointly affect adoption rates of tracking technology in both clinical and supply chain use contexts, which is essential for developing intelligent infrastructure for smart hospital systems. This study informs researchers, health care providers, and policy makers that hospital characteristics, locations, and governance structures have different impacts on the adoption of tracking technologies for clinical and supply chain use and on health resource disparities among hospitals of different sizes, locations, and governance structures.

## Introduction

### Background

The extensive adoption of innovative tracking technologies has left almost no industry behind. Owing to strict health care laws, regulations, and policies, the health care industry has made great strides in the area, with a growing number of hospitals in the United States and worldwide beginning to reap the benefits of tracking technologies involved in, for example, optimizing health care processes, minimizing waste and human errors, and enhancing operational efficiency [1,2]. Upon approval, tracking technologies can be applied to enable health care providers to develop technology infrastructure in hospitals, resulting in greater efficiency in locating medications, patients, and staff in clinical processes and in tracking equipment, enhancing information sharing, and facilitating operations in the supply chain management process [1-5]. One of the main drivers in adopting tracking technologies is the meaningful use incentive program, which provides financial incentives for health care providers who use *certified* health technologies to meet a set of use criteria specified by the Centers for Medicare and Medicaid Services [6]. This program comprises 3 stages: stage 1 focuses on data capture and sharing, stage 2 relates to advanced clinical processes such as using tracking technologies for medication, and stage 3 concerns improved outcomes. The focus of our study is stage 2, where the adoption rate of tracking technologies has been increasing, given that the use of autotracking technologies to improve clinical processes has been one of the meaningful use core measures of stage 2, since 2012, effective in 2014 [7].

Of several applied instances in the field of tracking technology, barcodes and radio-frequency identification (RFID) are the most widely adopted tracking technologies [8]. Barcoding was introduced and used successfully in the health care industry 2 decades ago [7,9]. Linear or complex barcode technologies can encode patient, medicine, and asset information [9]. Unlike barcodes, which can only be read in *line of sight*, RFID has the advantage of using radio waves for automatic object identification, asset tracking, and data collection and transfer [10,11]. The implementation of RFID in the health care industry has been relatively recent and has become one of the major technological advancements in the future development of the health care sector [11,12]. For example, RFID can be applied with photosensors for smart hospitals to develop intelligent infrastructure, enabling better interactions between health care providers and patients and allowing more transparent and timely access to medical data [10]. As such, tracking technologies are essential for developing an intelligent infrastructure for current smart hospital systems, meaning that one of the goals of this study is to understand the factors involved in adopting tracking technologies (eg, barcoding and RFID).

Because of the appealing potential of tracking technologies to automate data, improve security, reduce counterfeiting and theft, and expedite and optimize clinical processes and supply chain management in the health care industry, their adoption in clinical and supply chain uses has been significantly outpaced by other widely adopted health technologies such as electronic health record systems [8,10-14]. Studies on the factors involved in health technology adoption are extensive in the medical, health informatics, and information systems literature, with hospital characteristics such as hospital size, teaching status, payer mix, accreditation status, ownership, and hospital affiliation being well documented as key factors influencing the implementation levels of various health technologies [15-19]. Tracking technologies such as RFID and barcodes have been carefully examined, as they possess the potential to automate and streamline processes in health care intelligence to improve clinical decision-making, patient care, and health care ecosystems for more accurate and efficient operations [20-22]. As such, tracking technologies, if adopted, can expedite and optimize clinical processes and health outcomes by tracking patients, health care workers, and hospital assets in real time [12,21,22], minimizing man-made mistakes and negligence [3,23-27] and delivering accurate information to health care systems, thereby unleashing the untapped benefits of digital innovations [3,22]. Together, they provide a solid foundation for efficient and effective health care practices, leading to more intelligent health care systems and operations [1,28]. Given the critical role and promising outlook of tracking technologies in health care, it is imperative to understand the key factors driving their adoption, which are already readily embedded in existing health care systems. However, to the best of our knowledge, no previous studies have systematically examined the critical factors that influence tracking adoption in clinical and supply chain use contexts, meaning that immediate investigation is required.

Within the umbrella of digital innovation, tracking technologies share some similarities, such as adopting digital features with other health information technologies (HITs). Nevertheless, they display a range of unique and distinctive characteristics that require thorough legal, clinical, and practical examination before adoption. First, unlike other HITs, concerns over privacy and security related to the use of tracking technologies are more prevalent and substantial [28], in that data associated with tracking technologies are under tight restrictions and protection, as per the Health Insurance Portability and Accountability Act (eg, patient information including name, medical record number, and date of birth) [23]. In this regard, other factors such as time effects must be considered in the adoption process; understandably, little research has been undertaken to explore this because of the lack of available data. Second, previous studies largely considered HITs in the context of clinical use, incorporating technologies primarily used to aid clinical-focused processes, including capture, storage, and processing of clinical information, such as doctors’ notes, patient records, and test results, as well as auxiliary systems for order entry and decision support [29]. In contrast, other HIT use contexts, such as HITs in supply chains, are generally offstage. Tracking technologies not only augment and optimize the capabilities of HITs in clinical use, speeding up clinical processes, for example, and facilitating supply chain management processes to be more cost-effective but also reduce unnecessary waste [28]. Therefore, the key factors that affect the adoption of HITs in the clinical process are likely to be distinctive from those contributing to the adoption of tracking technologies in supply chains, inviting further but separate investigations. For example, the type of hospital affiliation can impact supply chain use but not necessarily clinical use. Tracking technologies are also favorably envisioned as embedded in smart hospitals and Internet of Things–based hospitals as part of common intelligent health care initiatives to optimize health care processes, improve operational efficiency, and enhance safety for both patients and medical practitioners, particularly benefiting disadvantaged groups such as people who are disabled and older adults [11,30-33]. For instance, an assisted living system can locate and track people who are disabled and older adults, alert caregivers in real time in unexpected situations, and support daily activities for people who are disabled and older adults, including reminding them to take their daily medications safely [10,11,34]. Understanding the potential factors involved in the adoption of tracking technology pinpoints the complexities incorporated in taking full advantage of digital innovations, ranging from resource distribution to managerial, operational, and clinical practices central to achieving equitable and smart health care.

### Objectives

One innovation in hospital management over the past few decades is strategic reconfiguration, which consolidates individual, unaffiliated hospitals into multihospital systems [35]. Previous studies have emphasized the importance of hospital systems in health technology adoption—hospitals affiliated with a health system can allocate more resources, improve coordination, increase market shares, and embrace greater financial performance and are more likely to adopt HITs, such as electronic medical record [19,35,36]. Hospitals affiliated with various health system types (eg, centralized vs independent health systems) may have different governance structures, indicating underlying mechanisms to ensure order in workflow management, including how work arrangements are structured (eg, structured within trustful networks, within various governance hierarchies, or as impersonal exchanges), and may have different service types (eg, centralized health systems with high levels of centralization of health services and decentralized health systems with high levels of decentralization of hospital services) [35]. This differentiation enables varying hospital work arrangements and health service types because of different governance structures and further affects technology adoption decisions. However, the general influence of governance structure on technology adoption in different use contexts in hospitals remains limited, and to the best of our knowledge, no study has examined this issue systematically based on a comprehensive data set. Our study, therefore, aims to extend the current understanding by identifying the relationship between hospital governance structure and tracking technology adoption.

Of the very limited number of quantitative studies previously undertaken to explore factors involved in the adoption of tracking technology in hospitals in the United States, Dey et al [28] conducted an empirical study in 2010 of 86 US hospitals, finding that both organizational and technological factors affect decisions to adopt RFID, whereas environmental factors such as uncertainty in a competitive market do not. Uy et al [7] examined the adoption trends of barcodes and RFID technologies with a larger data set of >5400 US hospitals from 2008 to 2012 and found that, in 2012, the adoption rate of barcodes for medication administration reached 58.1%, whereas adoption of RFIDs remained at 1.87%. Both witnessed their highest growth in adoption of medication administration in this period, compared with laboratory, pharmacy, and radiology use [7]. Of previous review studies on adoption of tracking technology, Wamba [4] conducted a comprehensive review of 22 articles published in the Journal of Medical Systems between 1997 and 2011 relating to application areas, types of benefits, and types of issues linked to RFID technology adoption. This review paper pointed out that the most highly published application area was that of patient management, the most widely discussed benefit was efficiency gain, and the most examined issues were data management, security, and privacy. These studies, however, were often limited by a small sample size [28] and considered only the early adoption period for tracking technologies (eg, 1997 to 2011 [4] and 2008 to 2012 [7]), reinforcing the notable gap in the longitudinal understanding of such tracking technologies adopted in health care for both clinical and supply chain use [1] and calling for more academic attention and further investigation.

This study, therefore, took the lead as the first longitudinal research study to empirically examine the different factors associated with the adoption of tracking technologies in different use contexts with more recent US hospital data sets. This was the first study to examine the impact of governance structure types on technology adoption in different use contexts in hospitals. Because of the complex nature of health care settings, we differentiate among the factors that influence the adoption of tracking technologies in the clinical and supply chain use contexts. Extant literature suggests that larger, urban, nonprofit, and teaching hospitals tend to possess more advanced resources, admit more complex patients with severe illnesses or multiple chronic conditions, and need to manage more complicated clinical workflows. When appropriate, these hospitals might implement a higher level of tracking technology to facilitate their clinical processes [37-39]. In response, we examined whether hospital characteristics and locations would impact tracking technology adoption in a clinical context. Existing studies also posit that the more centralized the health system to which the hospital is affiliated, the more likely it is that the hospital has more centrally organized service delivery with higher incentives and resources such as revenue and personnel to enhance supply chain efficiency using tracking technologies [19,40]. As a result, we examined whether governance structure types would affect tracking technology adoption in the context of supply chain use. In summary, with a large US hospital-level longitudinal data set, we aim to (1) compare critical factors associated with the adoption of tracking technologies for clinical and supply chain uses and (2) examine how governance structure types affect the adoption of tracking technologies in different use contexts in hospitals.

## Methods

### Data and Sample

The data sets used in this study are obtained from 3 sources: the American Hospital Association’s (AHA) annual surveys, the AHA’s information technology (IT) supplemental files, and the US Bureau of Economic Analysis website. First, we collected data from the AHA’s annual surveys to identify hospital characteristics and obtain health system data. Second, we used the AHA’s IT supplemental files to capture the tracking technology implementation data. Third, we used data from the US Bureau of Economic Analysis website to obtain gross domestic product (GDP) per capita information [41]. The period from 2012 to 2015 was selected because, from 2012, the Centers for Medicare and Medicaid Services required hospitals to initiate tracking of medications using tracking technologies, including RFID or barcoding as part of meaningful use core measures [7]. Our final data set is a longitudinal one containing 3623 general medical and surgical hospitals from 50 states in the United States, from 2012 to 2015, of which 74.19% (2688/3623) of hospitals were observed at least twice.

### Dependent Variables

The 2 dependent variables used in this study are tracking technology adoption for clinical use and supply chain use. We constructed tracking technology in a clinical use context by counting the number of technologies fully implemented and replacing paper record functionality at a hospital, an approach widely used in information systems and health care literature [29,42]. Implementation levels of tracking technology for each itemized technology are measured on a 6-point scale, where 1 indicates *fully implemented across all units*, 2 to 5 indicates *partially implemented* at different levels, and 6 indicates *not in place and not considering implementing.* To calculate the implementation level for each technology function, we applied a coding scheme to separate full implementation—technology has completely replaced paper record functionality—from partial or no implementation. We retained the original coding of 1 as 1 and then coded the responses between 2 and 6 as 0. There are 4 tracking technology functions in the clinical use context: medication administration, patient verification, caregiver verification, and pharmacy verification. Therefore, the resultant tracking technology implementation level in a clinical use context ranged from 0 to 4. On this scale, 0, 1, 2, 3, and 4 indicate that the hospital fully implemented none, one, two, three, and four of the four tracking technologies, respectively. We then applied a binary variable to code completely implemented tracking technology in a clinical context across all 4 technology functions, for which 1 indicates fully implemented across all *four* technology functions and 0, otherwise. We also used a binary variable to code the tracking technology for supply chain management, as there is only 1 technology unit in this variable, for which 1 indicates fully implemented and 0, otherwise. For example, if a hospital has completely digitalized its tracking process using tracking technologies for clinical use, including medication administration, patient verification, caregiver verification, pharmacy verification, and supply chain management, the hospital’s clinical and supply use adoption will be coded as 1.

### Independent Variables

We included 3 sets of independent explanatory variables. The first set of variables was related to hospital characteristics, such as hospital size, ownership, and teaching status. *Hospital size* was measured based on the number of staffed beds. *Hospital ownership status* was coded as a binary variable denoting whether the hospital was a for-profit hospital. *Teaching status* was also coded as a binary variable, where 1 indicated a teaching hospital and 0, otherwise. We defined teaching hospitals as members of the Council of Teaching Hospitals of the Association of American Medical Colleges. The second set included the hospital location variables. *Hospital location* was measured using 3 dummy variables: metropolitan, micropolitan, and rural regions. We also measured the *state economic condition* where the hospital is located because previous studies found that per capita GDP plays an important role in technology adoption and use [43,44]. Thus, we assume that such a condition would be linked to the adoption of digital innovations in health care, which is worth examining in the context of tracking technology. Per capita GDP was measured using GDP per capita per state. We first ranked hospitals from high to low based on their state GDP per capita. We then coded hospitals belonging to the first half as *economic leading state* and those in the second half as *economic leveling state.* The third set comprises hospital governance structures because previous research indicates that governance structure is significantly associated with technology adoption [19,40]. *Governance structure* is measured according to whether the hospital is affiliated with a health system and, if so, the level of centralization in multihospital systems. Centralized health systems have unified asset ownership of affiliated hospitals and offer an array of products and services [45]. As in previous research [19,40], we applied 5 dummy variables to measure governance structure based on the diversification of health services and products and centralization of authority across health systems (out of health system as the base category) [45]. These variables define whether hospitals are (1) in centralized health systems with high levels of centralization of hospital service delivery, physician arrangements, and insurance product development; (2) in health systems with highly centralized physician arrangements and insurance product development; (3) in moderately centralized health systems with both centralized and decentralized activities for hospital services, physician arrangements, and insurance product development; (4) in decentralized health systems with a high degree of decentralization of hospital services, physician arrangements, and insurance product development; and (5) in independent hospital systems with limited differentiation among hospital services, physician arrangements, and insurance product development.

### Statistical Analysis

To examine the factors involved in the adoption of tracking technology in both clinical and supply chain use contexts in US hospitals, we used a mixed effects model using a population approach. This model is an extension of the simple fixed effects modeling to account for both fixed and random effects. This is particularly useful when data violate the independence assumption that arises from a hierarchical structure. For example, in this study, there were 2 levels: between hospitals (level 1) and within hospitals (level 2). As the data records for this study were collected from 3623 hospitals over 4 years, the source of variability in the observations can be attributed to either within-hospital or between-hospital effects. Repeated observations over the years from the same hospital are subject to hospital-level time-invariant unobserved effects, as within a given hospital, records are more similar. The units sampled at the highest level (ie, hospitals in this study) were independent. As our 2 dependent variables—tracking technology adoption for clinical use and tracking technology adoption for supply chain use—are binary variables, we developed a mixed effects population logistic regression model to examine the relationships among the adoption of tracking technologies (ie, clinical use vs supply chain use), hospital characteristics, and governance structure with the adjustment of time effect. Nonlinear mixed effects modeling software (NONMEM, version 7.5.0; ICON Development Solutions) was used for the modeling [46]. The Laplace estimation method was applied for parameter estimation. Perl Speaks NONMEM (PsN 4.8.0; Department of Pharmacy, Uppsala University) was used for model diagnostics and R (version 3.5.3) was used for data exploration before modeling and postprocessing of the results [29].

Initially, correlations among the covariates were explored. Exploratory graphical and statistical evaluations were performed to identify the relationship between estimated individual random effects and covariates. ANOVA tests for categorical covariates and linear regression for continuous covariates were used to identify possible univariate covariate relationships at *P*<.05. Only after statistically significant covariates were identified was the association between relevant hospital covariates and tracking technology adoption parameters further evaluated using a forward inclusion and backward elimination strategy, with model selection carried out using a log likelihood ratio test at an acceptance *P* value of .01 (a decrease in objective function value>6.63) in the forward step and a *P* value of .001 (an increase in objective function value >10.83) in the backward step. The final selection of relevant covariates was based on statistical evidence and health technology knowledge and interpretation. The derived model was further refined based on model convergence, parameter precision and impact of the covariate effect. The predictive performance of the final population logistic regression model was evaluated using visual predictive check (VPC) plots. Plots of observed data distributions were compared with simulated distributions to demonstrate the model’s ability to adequately predict data on which the model is based. VPCs were based on 1000 simulations and stratified by the covariates of potential interest.

## Results

### Overview

A total of 3623 hospitals in 50 states in the United States, from 2012 to 2015, were included in this study (the complete list of hospitals can be accessed in Multimedia Appendix 1). Of these 3623 hospitals, 928 (25.61%) were in rural areas, 3133 (86.48%) were nonprofit hospitals, 223 (6.16%) were teaching hospitals, and 2158 (59.56%) were affiliated with health systems, and the mean total of the number of beds was 174 (SD 201). Detailed demographics of the included hospitals are listed in Table 1, and Table 2 presents the results of the adoption of tracking technologies over time. The AHA IT supplement survey specifies fully implemented as the status of technology that has completely replaced paper record functionality. In this regard, from 2012 to 2015, as per the data set, the proportion of hospitals that have fully adopted tracking technologies in digitalized medication administration, patient verification, caregiver verification, and pharmacy verification in a clinical use context increased from 36.34% (782/2152) to 54.63% (1316/2409), whereas the proportion of hospitals that have fully adopted tracking technologies to digitalize supply chain management increased from 28.58% (615/2152) to 41.3% (995/2409), demonstrating that the tracking functionality for both clinical use and supply chain use has been increasingly digitalized in this period.

**Table 1:** Demographic information from the included hospitals (N=3623).

Demographics		Overall
**Location, n (%)**		
	Metro	2019 (55.72)
	Micro	676 (18.65)
	Rural	928 (25.61)
**Profit, n (%)**		
	Not-for-profit	3133 (86.47)
	For-profit	490 (13.52)
**Teaching hospital, n (%)**		
	Yes	223 (6.15)
	No	3400 (93.84)
**State economic condition^a^, n (%)**		
	Economic leveling state	1753 (48.38)
	Economic leading state	1870 (51.61)
**Governance structure: type of hospital affiliation (HS)^b^, n (%)**		
	Centralized HS	310 (8.55)
	Centralized physician and insurance HS	54 (1.49)
	Moderately centralized HS	276 (7.61)
	Decentralized HS	1419 (39.16)
	Independent HS	99 (2.73)
	Within HS	2158 (59.56)
	Out of HS	1465 (40.43)
Total bed count, mean (SD)		174 (201)

^a^Economic leading state: top 25 states in gross domestic product per capita; economic leveling state: last 25 states in gross domestic product per capita.

^b^HS: health system.

**Table 2:** Adoption of tracking technologies in the United States from 2012 to 2015.

Usage		Tracking technologies year, n (%)			
		2012 (N=2152)	2013 (N=2012)	2014 (N=2277)	2015 (N=2409)
**Clinical use**					
	Fully implemented	782 (36.33)	892 (44.33)	1190 (52.26)	1316 (54.62)
	Not fully implemented	1370 (63.66)	1120 (55.66)	1087 (47.73)	1093 (45.37)
**Supply**					
	Fully implemented	615 (28.57)	746 (37.07)	909 (39.92)	995 (41.3)
	Not fully implemented	1537 (71.42)	1266 (62.92)	1368 (60.07)	1414 (58.69)

### Tracking Technologies for Clinical Use

**Figure 1:**
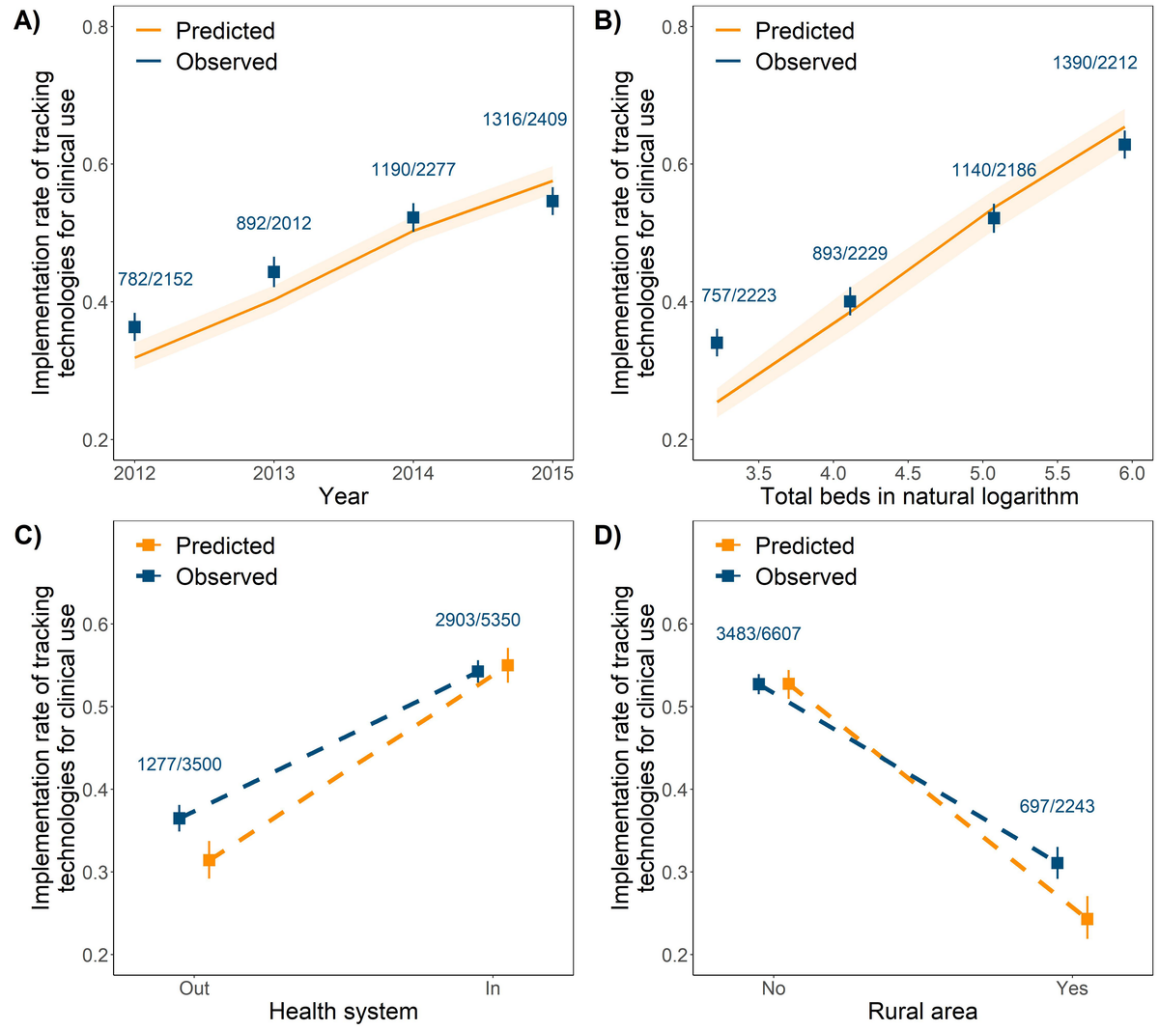
Visual predictive check plots of final population logistic regression model for the adoption of tracking technologies for clinical use over time. (A) the influence of time on the implementation rate of tracking technologies for clinical use; (B) the influence of total beds on the implementation rate of tracking technologies for clinical use; (C) the influence of health system on the implementation rate of tracking technologies for clinical use; (D) the influence of location (in the rural area or not) on the implementation rate of tracking technologies for clinical use. The blue dots show observed implementation rate; the blue error bars indicate a 95% CI in the observed implementation rate; the yellow dots and yellow solid lines show the median implementation rate from model prediction; the yellow error bars and the yellow area indicate a 95% prediction interval for the implementation rate.

**Figure 2:**
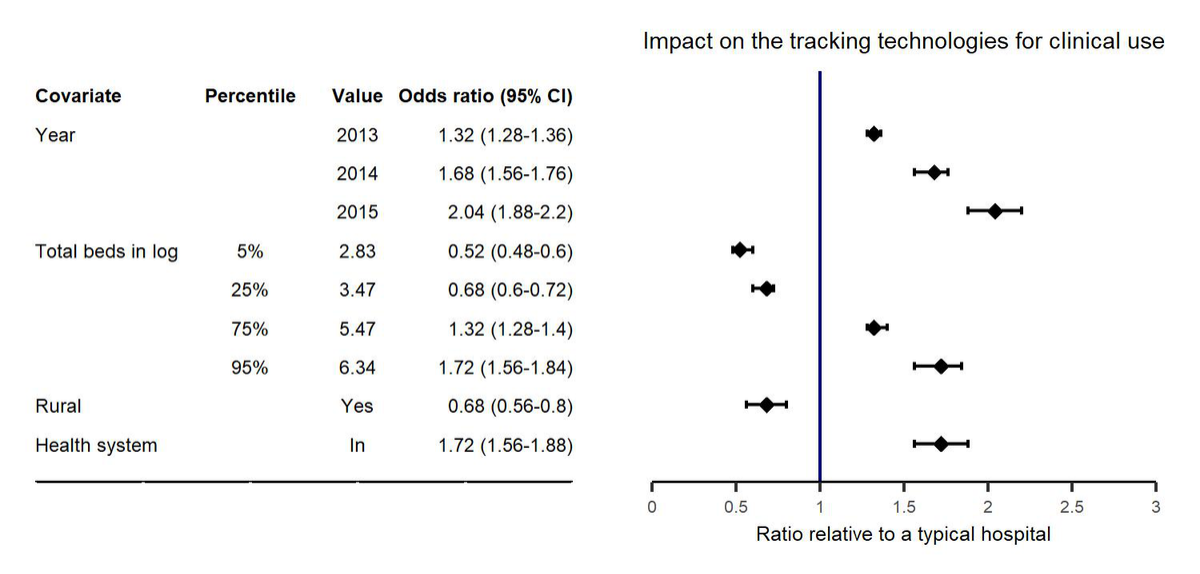
Forrest plot of covariate effects on the implementation rate of tracking technologies for clinical use. The solid vertical line corresponds to a ratio of 1 and represents a typical hospital. Points and whiskers represent the estimate and 95% CI, respectively. A typical hospital is defined as a hospital with a total of 101 beds, not part of a health system, and not in a rural area in 2012.

As shown in the VPC plots (Figure 1), the mixed effects population logistic regression model developed could well describe the adoption of tracking technologies for clinical use. All the population parameters for a typical hospital (defined as a hospital with 101 beds, not affiliated to a health system, and not in a rural area) were precisely estimated: the intercept was estimated to be −1.08 (relative SE 8%), and the slope was estimated to be 0.369 (relative SE 8%; Table 3). The total beds in natural logarithm, rural locations, and health systems were statistically significant covariates on the intercept. The relative univariate effects of total beds, rural locations, and health systems on the implementation rate of tracking technologies for clinical use are summarized as a forest plot in Figure 2.

The model developed has the potential to predict the increasing trend in the implementation rate of tracking technologies in clinical use over a period of years (Figure 1A). A positive relationship was identified with hospital size (reflected by the total number of beds; Figure 1B). Similarly, the implementation rate increased by a median of 1.7-fold for hospitals affiliated with the health system relative to those that were not affiliated (Figure 1C and Figure 2). These results imply the influence of hospital infrastructure (both physical and organizational structures) on the adoption of tracking technologies in clinical use. Meanwhile, the implementation rate decreased by a median of 32% in hospitals located in rural areas relative to those in urban areas, showing clear evidence of location disparity (Figure 1D and Figure 2).

**Table 3:** Parameter estimates of final population logistic regression model for the adoption of tracking technologies for clinical use.

			Estimate (relative SE; %)
**Fixed effects**			
	Intercept	−1.08 (8)	
	Time effect	0.369 (8)	
	Log total bed	0.452 (10)	
	Rural area	−0.535 (21)	
	Health system	0.79 (11)	
**Random effects**			
	Intercept	2.55 (8)	
	Time effect	0.11 (47)	



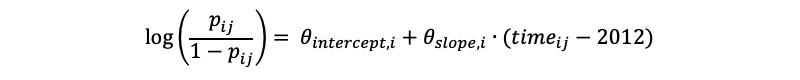





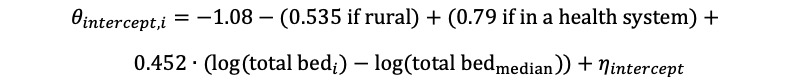





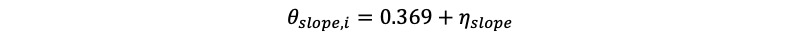





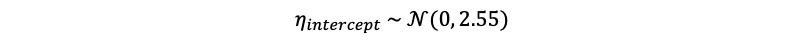





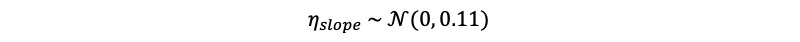



### Tracking Technologies for Supply Chain Use

**Figure 3:**
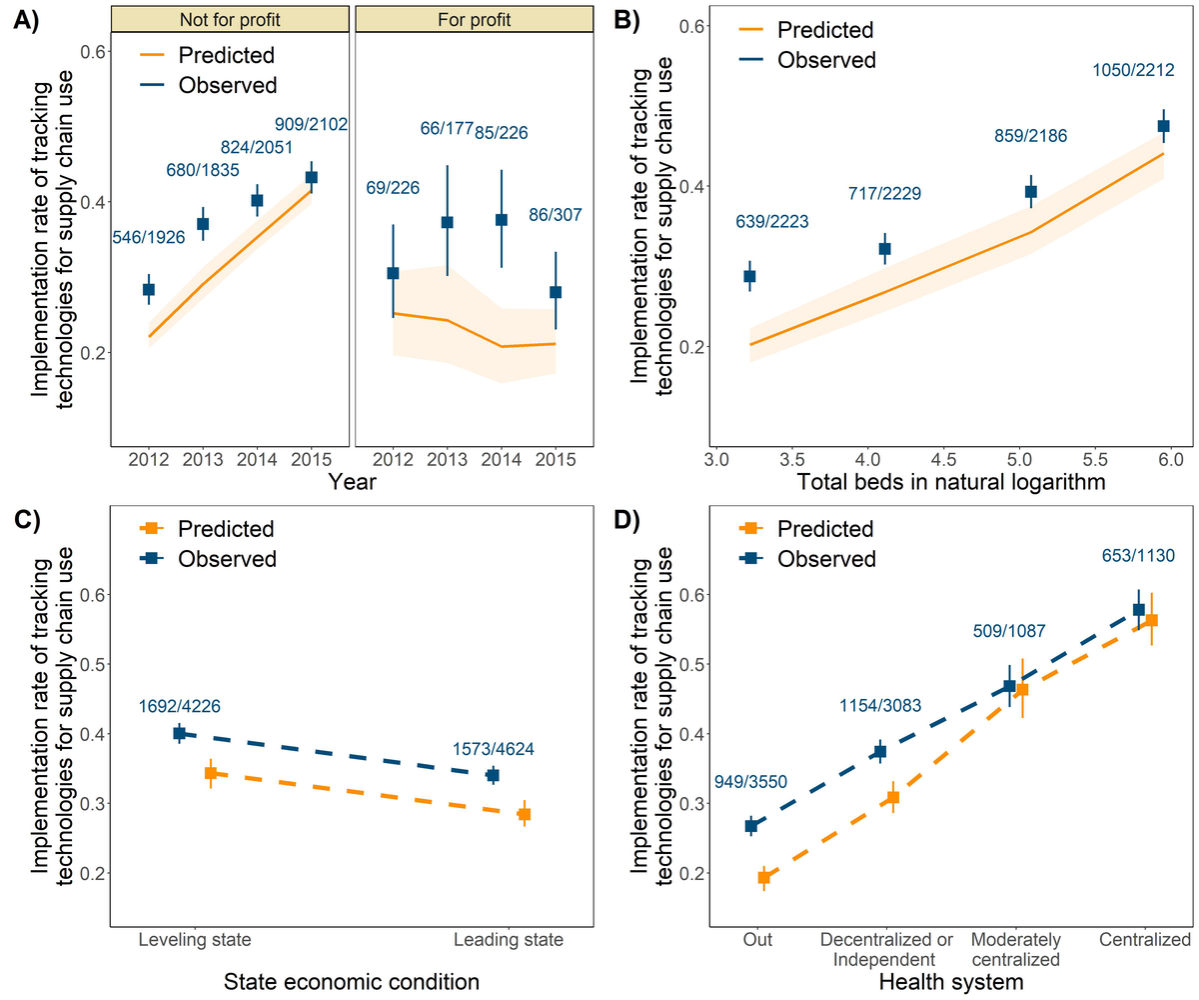
Visual predictive check plots of final population logistic regression model for the adoption of tracking technologies for supply chain use over time. (A) the influence of time on the implementation rate of tracking technologies for supply chain use; (B) the influence of total beds on the implementation rate of tracking technologies for supply chain use; (C) the influence of state economic condition on the implementation rate of tracking technologies for supply chain use; (D) the influence of health system on the implementation rate of tracking technologies for supply chain use. The blue dots show observed implementation rate; the blue error bars indicate a 95% CI in the observed implementation rate; the yellow dots and yellow solid lines show the median implementation rate from model prediction; the yellow error bars and the yellow area indicate a 95% prediction interval in the implementation rate.

**Figure 4:**
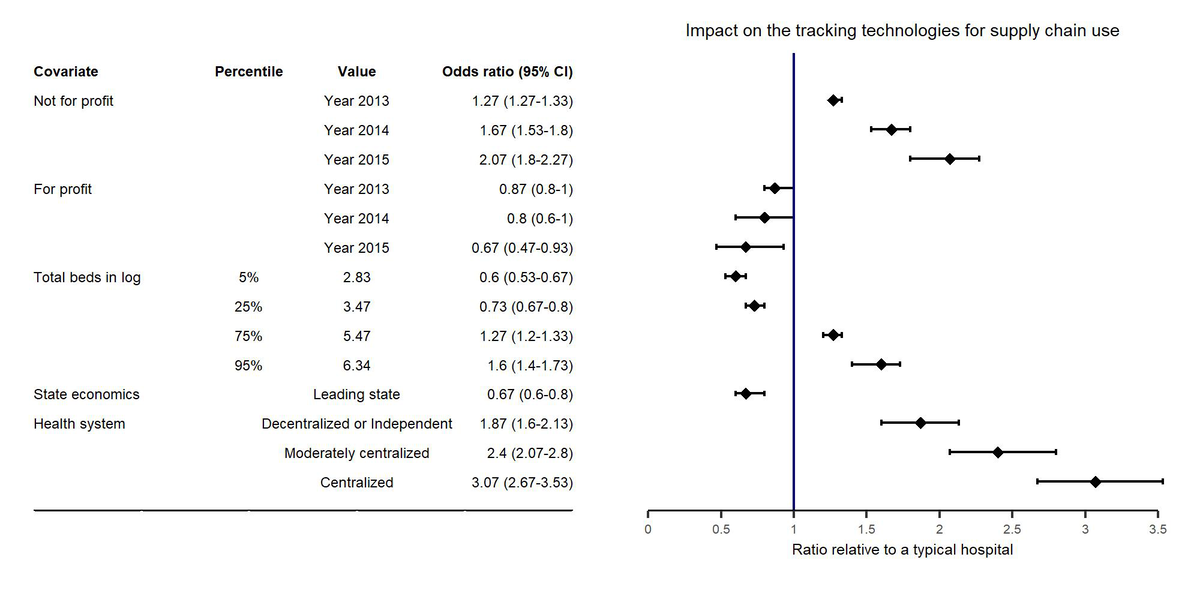
Forrest plot of covariate effects on implementation rate of tracking technologies for supply chain use. The solid vertical line corresponds to a ratio of 1 and represents a typical hospital. Points and whiskers represent the estimate and 95% CI, respectively. A typical hospital is defined as a not-for-profit hospital with a total of 101 beds, not part of a health system, and in an economic leveling state in 2012.

As shown in the VPC plots (Figure 3), the mixed effects population logistic regression model developed could well describe the adoption of tracking technologies for supply chain use. All population parameters for a typical hospital (defined as a nonprofit hospital with 101 beds and not affiliated with a health system in an economic leveling state) were precisely estimated: the intercept was estimated to be −1.72 (relative SE 6%), and the slope was estimated to be 0.3 (relative SE 10%; Table 4). Total beds in natural logarithm, state economic condition, and affiliation to a health system were found to be statistically significant covariates on the intercept, and profit status was found to be a statistically significant covariate on the slope. The relative univariate effects of total beds, state economic condition, and type of hospital affiliation on the implementation rate of tracking technologies in supply chain use are summarized as a forest plot in Figure 4.

The model developed can also predict the increasing trend in the implementation rate of tracking technologies for supply chain use over a period of 4 years in not-for-profit hospitals, as well as stagnation in development among hospitals running for profit (Figure 3A). This indicates that for-profit hospitals are more reluctant to implement these new technologies. The implementation rate of tracking technologies for supply chain use grew in parallel with increasing hospital size (as reflected by the total number of beds in the hospital; Figure 3B). Furthermore, implementation rates increased as hospitals affiliated with a more centralized health system—1.9-fold increase (odds ratio [OR] 1.87, 95% CI 1.60-2.13) for decentralized or independent hospitals, 2.4-fold increase (OR 2.40, 95% CI 2.07-2.80) for moderately centralized health systems, and 3.1-fold increase for centralized health systems (OR 3.07, 95% CI 2.67-3.53), compared with hospitals not affiliated with a health system (Figure 3D and Figure 4). When compared with tracking technologies for clinical use, these results demonstrate a similar impact of hospital infrastructure on the adoption of tracking technologies for supply chain use: hospitals with better infrastructure tend to be more responsive in adopting tracking technologies. Surprisingly, the implementation rate decreased by a median of 30% in hospitals in economic leading states relative to those in economic leveling states (Figures 3C and Figure 4).

**Table 4:** Parameter estimates of final population logistic regression model for the adoption of tracking technologies for supply chain use.

			Estimate (relative SE; %)
**Fixed effects**			
	Intercept	−1.72 (6)	
	Time effect	0.3 (10)	
	Log total beds	0.321 (12)	
	Economic leading state	−0.428 (20)	
	Centralized HS^a^	1.57 (9)	
	Moderately centralized HS	1.16 (11)	
	Decentralized or independent HS	0.772 (13)	
	Run for-profit effect on time effect	−1.48 (15)	
**Random effects**			
	Intercept	3.22 (8)	
	Time effect	—^b^	

^a^HS: health system.

^b^Data does not support the inclusion of random effect on time effect.



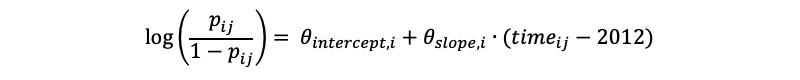





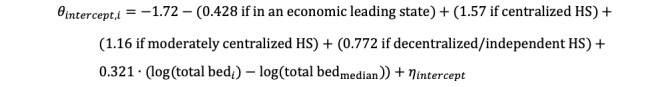





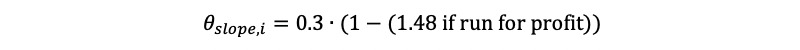





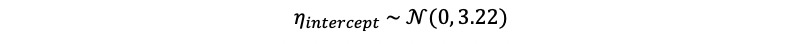



## Discussion

### Principal Findings

With a large US hospital-level longitudinal data set, we observed that, from 2012 to 2015, the proportion of hospitals in which tracking technologies were fully implemented for clinical use increased from 36.34% (782/2152) to 54.63% (1316/2409) and for supply chain use increased from 28.58% (615/2152) to 41.3% (995/2409). We found that larger hospitals were more likely to fully adopt tracking technologies in both clinical and supply chain use contexts, indicating health resource disparities among hospitals of different sizes. We also discovered that adoption factors affect the clinical and supply chain use contexts differently. In the clinical use context, compared with hospitals located in urban areas, hospitals in rural areas (OR 0.68, 95% CI 0.56-0.80) are less likely to fully adopt tracking technologies, showing evidence of location disparity. In the context of supply chain use, the type of governance structure influences tracking technology adoption. Compared with hospitals not affiliated with a health system, implementation rates increased as hospitals affiliated with a more centralized health system—1.9-fold increase (OR 1.87, 95% CI 1.60-2.13) for decentralized or independent hospitals, 2.4-fold increase (OR 2.40, 95% CI 2.07-2.80) for moderately centralized health systems, and 3.1-fold increase for centralized health systems (OR 3.07, 95% CI 2.67-3.53).

### Comparison With Previous Work

Given that studies on the adoption of tracking technologies have lagged in general health technology adoption studies, and studies undertaken are either limited by a small sample size or subject to early adoption periods, we attempted to fill this gap by applying a census data set from 2012 to 2015 to examine the factors involved in tracking technology adoption in both clinical and supply chain use contexts. Using mixed effects population logistic regression models, we identified several hospital characteristics and governance structure factors associated with tracking technology adoption. Consistent with previous studies on the impact of hospital size on technology adoption [15,47], our results show that larger hospitals are more likely to adopt tracking technologies in both the clinical and supply chain use contexts. In addition to considering hospital size, we found that hospitals in health systems are more likely to adopt tracking technologies in both clinical and supply chain use contexts. One reason for this is that tracking technology implementation cannot be accomplished in a single stroke. It requires the integration of tags, readers, networks, databases, systems, and expertise from different domains including RFID and barcode technology, medicine, information systems development, telecommunications, and systems integration [44]. Tracking technology is also part of the hospital technology infrastructure because it allows hospitals to capture, store, and streamline data and processes and can be integrated with other HITs such as electronic health records [48]. As infrastructure technology, the benefit of tracking technology adoption may only be realized in the long term. Thus, large hospitals or those within health systems urgently need to manage more complex patients with multiple chronic conditions with sufficient resources and capabilities to implement tracking technology and process large volumes of real-time data generated by the tracking technology.

In the context of clinical use, our results supplement existing studies with additional findings, identifying that rural hospitals are less likely to adopt tracking technologies. One possible reason is that, in contrast to hospitals located in metropolitan and micropolitan areas, those in rural areas tend to accept patients with less severe and less complicated diseases, which are more easily diagnosed and treated by local health care providers, thus requiring less sophisticated technology for clinical use [38]. Another possible reason is that rural hospitals may have fewer available resources to promote and implement new HIT functions. For example, urban and suburban hospitals are found to have wide access to experienced technical support staff to assist with the implementation process for new HIT functions, resulting in more gains from the adoption of new HIT functions [49], whereas such high-caliber personnel may not be readily available for rural hospitals.

In the context of supply chain use, our results show that compared with not-for-profit hospitals, for-profit hospitals are less likely to adopt tracking technologies. Our results, shown in Figure 3A, indicate that the implementation rate of tracking technologies for for-profit hospitals increases and then decreases (inverted U-shaped implementation rate) over time but increases over time for not-for-profit hospitals. One possible reason is that for-profit hospitals tend to pursue a high return on investments and thus often invest more in profitable services and avoid less profitable investments [50]. Given that supply chain management is indirectly related to hospital revenue, the use of tracking technologies in supply chains may not be prioritized in their investment lists against budgets. Thus, only for-profit hospitals that are highly motivated to reap the benefits of tracking technology (eg, to enhance efficiency) and obtain financial incentives from meaningful use fully implemented tracking technologies immediately after the addition of the autotracking medication requirement as a core measure in meaningful use in 2012, explaining why the implementation rate increased sharply from 2012 to 2013 for for-profit hospitals and the acceleration rate of implementation decreased from 2013 to 2015 [6]. A surprising finding also emerges from research that hospitals in economic leading states are less likely to adopt tracking technologies for supply chain use. One plausible reason is that economic leading states are generally more populous and thus, these urban hospitals need to attend to larger numbers of patients, thereby putting their funding priorities more on efficiency enhancement for immediate patient treatment, involving featured clinical processes rather than managerial operations, such as supply chain management.

Our study also extends the current understanding of how governance structure influences technology adoption by identifying the relationship between governance structure types and tracking technology adoption. We revealed that hospitals affiliated with health systems are more likely to adopt tracking technologies for clinical use, whereas types of hospital affiliation do not affect the adoption of tracking technologies for clinical use. We also find that the type of hospital affiliation affects the adoption of tracking technologies for supply chain use—hospitals affiliated with more centralized health systems are more likely to adopt tracking technologies for supply chain use. Compared with other types of hospital affiliations (eg, decentralized or independent or moderately centralized), centralized systems provide a higher percentage of their services at the system level, making them more likely to have higher incentives to increase supply chain efficiency using tracking technologies and develop the long-term tracking technology–related infrastructure of smart hospitals [40]. In addition, tracking technologies adopted for supply chain use, compared with tracking technologies for clinical use, may be costlier, complicated, and take longer to implement, requiring systematic and strategic planning, implementation, and integration and a more centralized health system.

Overall, 3 implications are set out in our study for researchers, health care stakeholders, and policy makers. First, our study indicates that the context of technology use (ie, clinical use or supply chain use) influences the tracking of technology adoption. For example, we found that for supply chain use, governance structure types are important factors in the adoption of tracking technologies, but this is not the case for clinical use. In other words, there is no one-size-fits-all solution for adopting tracking technologies in the field of health care. When examining the impact of tracking technologies, practitioners, both academic and practical, should develop a holistic view of the adoption context and cannot assume that related factors can be generalized from other contexts. Health care practitioners who aspire to establish tracking technology–enabled (eg, RFID-enabled) smart hospitals, for example, are in favor of implementing tracking technologies for clinical use, facilitating information sharing, patient identification, and medical equipment tracking, and in supply chains to avoid drug counterfeiting and to enhance supply chain operations [11]. However, our study shows that the factors involved in the adoption of tracking technologies for clinical and supply chain use may be different. Understanding differences in adopting tracking technologies in various use contexts will help all hospitals involved in health care to plan and implement tracking technologies more strategically and avoid any possible pitfalls while maximizing their benefits from the outset. When tracking technologies are further leveraged in conjunction with other technologies, such as electronic health records, electronic data interchange technologies, mobile devices, and telehealth, caution over the context in question may still be relevant, suggesting that it is important for future studies to examine the different use contexts (eg, clinical and supply chain use) of tracking technologies, as highlighted in and demonstrated by our study.

Second, similar to initial studies that examine the effects of governance structure on longitudinal tracking technology adoption, our results suggest that the impact of governance structure types should be emphasized in technology adoption studies and that the underlying mechanisms require further investigation. For example, we identified that hospitals affiliated with more centralized health systems are more likely to adopt tracking technologies for supply chain use because of the centralized hospital structure settings, allowing resources to be prioritized and allocated to improve operational efficiency for more efficient and streamlined use, thus serving larger patient populations with personalized medicine. This feasibly occurs when systematic integration and synchronization for various solo practices are implemented in centralized smart hospital systems. Future studies are required to investigate the underlying mechanisms (ie, managerial support) linking technology adoption and governance structure and examine whether the findings of this study can be extended to other technology innovations.

Third, our results suggest that disparities may exist in health resources between hospitals of various sizes and governance structures. We found that larger hospitals and hospitals affiliated with health systems, especially more centralized health systems, are more likely to adopt tracking technologies. Compared with small and independent hospitals, these hospitals tend to have more human and financial resources to become the first adopters of advanced technologies. A potentially uneven distribution should be given ample attention before the trend becomes so established that it compounds the already sizable digital gap among different types of hospitals [51]. This is extremely important for smart hospital development, as tracking technologies can be applied as a technology infrastructure in smart ecosystem design. Given the increasingly important role of tracking technologies in transforming existing health care providers into smart hospitals, understanding the key factors involved in tracking technology adoption provides governments with evidence-based findings, supporting them to develop more feasible quantified health resources with an allocation scheme that promotes barcodes, RFID-enabled smart hospitals, and equitable health care. Thus, our study highlights the need for up-to-date government policies related to reasonable resource allocation for tracking technology implementation and its use in establishing and developing smart hospitals, including the use of tracking technologies in patient care, drug management, security and privacy, and tailored interventions from regulatory bodies or policy makers.

### Strengths

This study is the first longitudinal research to empirically examine the different factors associated with the adoption of tracking technologies in different use contexts. This is also the first study to examine the impact of governance structure types on technology adoption in different use contexts in hospitals. In doing so, we provided a census assessment and longitudinal overview of how hospital characteristics and governance structure are related to the adoption rates of tracking technology in both clinical and supply chain use contexts. This study informs researchers, health care providers, and policy makers that hospital characteristics, locations, and governance structures have different impacts on the adoption of tracking technologies for clinical and supply chain use and on health resource disparities among hospitals of different sizes and with different locations and governance structures. This study has important managerial implications for the development of smart hospitals using tracking technologies to establish their hospital infrastructure and practical implications for examining the impact of governance structure types on the adoption of other technologies in health contexts.

### Limitations and Future Directions

This study had some limitations. First, as comprehensive as the data set was, the timeframe was limited to the period from 2012 to 2015. Despite our rationale to address the scarcity of research into health care tracking technology by combing through details related to the issue of tracking technology adoption since its initial implementation in 2012 for the second stage of meaningful use, we caution that further development could have been in place as part of recent uptakes. Thus, it is necessary to conduct this research in conjunction with additional data. Second, we put in place 2 application scenarios to examine tracking technology in the clinical and supply chain use contexts. However, this examination has the potential for a more detailed focus on capturing additional particulars. For example, future research could examine the factors that influence the implementation of different clinical uses of tracking technologies, such as medication administration, patient verification, caregiver verification, and pharmacy verification.

### Conclusions

This study provides a census assessment of the adoption of both clinical and supply chain tracking technologies in US hospitals and offers a comprehensive overview of the hospital characteristics and governance structure associated with tracking technology adoption. From an academic perspective, this study unearths the staggered adoption of health tracking technology in hospitals in various categories, suggesting that hospital characteristics and governance structures have a significant impact on the implementation level and rate of tracking technology in clinical and supply chain use. It expands our understanding of digital innovations in health care, providing further evidence relating to tracking technology and outlining implications that can be leveraged from a managerial point of view. This study informs health care providers and policy makers of the possible guidance references that tailored policies should be in place to further promote the ongoing digital transformation in health care, as hospital characteristics and governance structures have different influences on the digitalization process. These outcomes can facilitate both academics and practitioners in putting forward future research to further reveal the nature and scope of tracking technology in developing smart hospitals and personalized health care in general.

## Appendix

Multimedia Appendix 1 Complete list of hospitals applied in this study.

## Abbreviations

AHA: American Hospital Association
GDP: gross domestic product
HIT: health information technology
IT: information technology
OR: odds ratio
RFID: radio-frequency identification
VPC: visual predictive check

## Data Availability

The data sets analyzed during this study are not publicly available owing to the restrictions of use from the American Hospital Association data use agreement but can be accessed with permission from the American Hospital Association [52].
